# A H_**2**_S Donor GYY4137 Exacerbates Cisplatin-Induced Nephrotoxicity in Mice

**DOI:** 10.1155/2016/8145785

**Published:** 2016-05-31

**Authors:** Mi Liu, Zhanjun Jia, Ying Sun, Aihua Zhang, Tianxin Yang

**Affiliations:** ^1^Institute of Hypertension, Sun Yat-Sen University School of Medicine, No. 74 Zhongshan 2nd Road, Science and Technology Building, 6th Floor, Guangzhou 510080, China; ^2^Department of Medicine and Veterans Affairs Medical Center, University of Utah, Salt Lake City, UT 84132, USA; ^3^Nanjing Key Laboratory of Pediatrics, Nanjing 210008, China

## Abstract

Accumulating evidence demonstrated that hydrogen sulfide (H_2_S) is highly involved in inflammation, oxidative stress, and apoptosis and contributes to the pathogenesis of kidney diseases. However, the role of H_2_S in cisplatin nephrotoxicity is still debatable. Here we investigated the effect of GYY4137, a novel slow-releasing H_2_S donor, on cisplatin nephrotoxicity in mice. Male C57BL/6 mice were pretreated with GYY4137 for 72 h prior to cisplatin injection. After cisplatin treatment for 72 h, mice developed obvious renal dysfunction and kidney injury as evidenced by elevated blood urea nitrogen (BUN) and histological damage. Consistently, these mice also showed increased proinflammatory cytokines such as TNF-*α*, IL-6, and IL-1*β* in circulation and/or kidney tissues. Meanwhile, circulating thiobarbituric aid-reactive substances (TBARS) and renal apoptotic indices including caspase-3, Bak, and Bax were all elevated. However, application of GYY4137 further aggravated renal dysfunction and kidney structural injury in line with promoted inflammation, oxidative stress, and apoptotic response following cisplatin treatment. Taken together, our results suggested that GYY4137 exacerbated cisplatin-induced nephrotoxicity in mice possibly through promoting inflammation, oxidative stress, and apoptotic response.

## 1. Introduction

*cis*-Diamminedichloroplatinum (cisplatin) is one of the most potent anticancer drugs and is widely used as the front-line therapy for the treatment of tumors of head, neck, lungs, and genitourinary tract [1]. However, the clinical application of cisplatin is limited by its serious adverse effects, particularly irreversible nephrotoxicity [2, 3], which occurred in about one-third of patients with cisplatin treatment [4]. Nephrotoxicity induced by cisplatin has been ascribed to several mechanisms, including inflammation, oxidative stress, and apoptosis [5, 6].

Hydrogen sulfide (H_2_S) is the third gasotransmitter besides nitric oxide (NO) and carbon monoxide (CO) and attracted more and more attention because of its pleiotropic physiological effects. Endogenous H_2_S is generated mainly from L-cysteine by two key enzymes named cystathionine *β*-synthase (CBS) and cystathionine *γ*-lyase (CSE) [7]. Recently, emphasis has been placed on investigating whether H_2_S pathway is involved in different physiological or pathological processes by endogenous or exogenous perturbation. Exogenous H_2_S donor is reported to have renoprotective effects in prolonged warm renal ischemia-reperfusion injury [8], obstructive nephropathy [9], and gentamicin-induced renal injury [10]. The dysregulation of endogenous CBS or CSE also contributes to kidney ischemia-reperfusion injury [11] and diabetic nephropathy [12].

By reviewing the literatures, there exist inconsistent results about the effect of H_2_S on cisplatin-induced nephrotoxicity. One study showed that the inhibition of endogenous H_2_S production by DL-propargylglycine (PAG) could reduce renal damage induced by cisplatin through the restriction of inflammation in Wistar rats [13]. Oppositely, Ahangarpour et al. demonstrated that exogenous H_2_S donor sodium hydrosulfide (NaHS) was protective via antioxidant property in cisplatin-treated rats [14]. To address these controversies, we employed morpholin-4-ium 4-methoxyphenyl (morpholino) phosphinodithioate (GYY4137), a novel slow releasing H_2_S donor [15], to investigate the role of exogenous H_2_S in cisplatin-induced nephrotoxicity and the underlying mechanisms.

## 2. Materials and Methods

### 2.1. Materials

Cisplatin was purchased from Sigma-Aldrich (St. Louis, MO, USA). GYY4137 was purchased from Cayman Chemical Company (Ann Arbor, MI, USA), which was dissolved in 1 : 1 mixture of dimethyl sulfoxide (DMSO) and polyethylene glycol (PEG).

### 2.2. Animal Experiments

Male C57BL/6 mice (Jackson laboratory) aged 8–10 weeks old were maintained on a standard rodent chow with free access to food and water and were kept on a 12 h (light) : 12 h (dark) cycle. The animals were divided into three groups: control (CTR; *n* = 5), cisplatin alone (cp; *n* = 10), and cisplatin plus GYY4137 (cp + GYY4137, *n* = 10). Cisplatin was freshly prepared in ultrapure water at a concentration of 2 mg/mL and used to treat the mice by a single intraperitoneal (i.p.) injection (20 mg/kg), in both cp and cp + GYY4137 groups. GYY4137 was dissolved in 1 : 1 mixture of DMSO and PEG. The mice were pretreated for 72 h with a mixture of DMSO and PEG (CTR and cp groups) or GYY4137 (cp + GYY4137 group) at a dose of 21 mg/kg/d via a microosmotic pump (DURECT Corporation, Cupertino, CA, USA). To figure out the effect of GYY4137 alone on kidney morphology and function, another experiment was designed by the application of GYY4137 or vehicle to the mice for 6 days (*n* = 5 in each group). Animals were sacrificed after 72 h treatment of cisplatin or 6-day GYY4137 administration. Blood and kidney tissues were harvested for further analysis. All protocols employing mice were conducted in accordance with the principles and guidance of Sun Yat-Sen University Institutional Animal Care and Use Committee.

### 2.3. Renal Function and Histology

Blood urinary nitrogen (BUN) was determined to assess renal function. For histology, kidneys were fixed in 4% paraformaldehyde and stained with periodic acid-Schiff (PAS). The tissue damage was indicated by tubular lysis, dilation, disruption, necrosis, and cast formation. The degree of tissue damage was scored according to the percentage of damaged tubules as previously described: 0, no damage; 1, <25%; 2, 25–50%; 3, 50–75%; and 4, >75% [16].

### 2.4. Enzyme Immunoassay

The plasma TNF-*α* level was determined by an enzyme immunoassay kit (catalog number 559732, BD OptEIA, BD Biosciences, San Jose, CA, USA) according to the manufacturer's instructions.

### 2.5. Measurement of TBARS

The measurement of plasma thiobarbituric acid-reactive substances (TBARS) was based on the formation of malondialdehyde by using a commercially available TBARS Assay Kit (catalog number 10009055, Cayman Chemical) according to the manufacturer's instructions.

### 2.6. Western Blot Analysis

Isolated tissues were homogenized in ice-cold isolation solution with cocktail. The protein concentration was determined by Coomassie reagent. Protein lysates were denatured at 100°C for 10 min, separated by SDS-polyacrylamide gel electrophoresis, and transferred onto PVDF membranes. The bolts were blocked with 5% nonfat dry milk for 1 h and then probed with primary antibodies directed to CBS (Santa Cruz), CSE (Abcam), or *β*-actin (Sigma-Aldrich) overnight at 4°C, washed three times with Tris-buffered saline (TBS) containing 0.1% v/v Tween-20, and incubated for 1 h at room temperature (RT) with secondary antibodies (goat anti-rabbit IgG and goat anti-mouse IgG, Santa Cruz). The immunoreactive bands were visualized using chemiluminescent reagent (Thermo Scientific) and exposed to X-ray film. Resulting blots were scanned and quantified using Image-Pro Plus 6.0 software.

### 2.7. Quantitative Real-Time PCR (qRT-PCR)

Total RNA was isolated using TRIzol (Invitrogen) and first-strand cDNAs were synthesized from 4 *μ*g of total RNAs in a 20 *μ*L reaction using Superscript (Invitrogen). The first-strand cDNAs served as the template for quantitative PCR (qPCR) performed in the Applied Biosystems 7900 Real Time PCR System using SYBR Green PCR reagent. Oligonucleotides were designed using Primer3 software (available at http://frodo.wi.mit.edu/primer3/) and the sequences are shown in Table 1. Cycling conditions were 95°C for 10 min, followed by 40 repeats of 95°C for 15 s, and 60°C for 1 min.

### 2.8. Statistical Analysis

All results were presented as means ± SE. The statistical analysis was performed using ANOVA followed by Bonferroni's test or unpaired Student's* t-*test with SPSS 13 statistical software. *p* < 0.05 was considered significant.

## 3. Results

### 3.1. GYY4137 Exacerbated Cisplatin-Induced Renal Dysfunction and Tubular Damage

**Figure 1:**
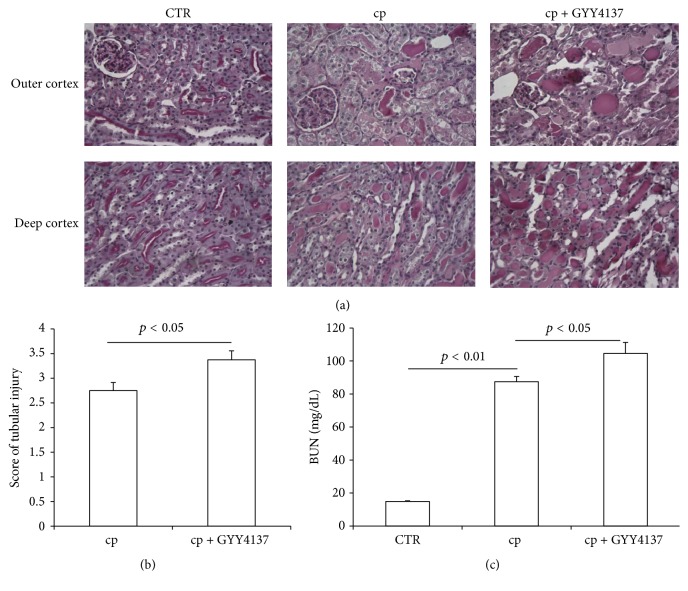
Effects of GYY4137 on renal function and tubular injury following cisplatin treatment. (a) Representative images of periodic acid-Schiff staining (×400) of kidneys. (b) Tubular injury score. (c) BUN levels. CTR: control, *n* = 5; cp: cisplatin, *n* = 10; and cp + GYY4137: cisplatin + GYY4137, *n* = 10. Data are means ± SE.

To evaluate the effects of GYY4137 on renal function in cisplatin-treated mice, we measured BUN level and found that BUN was robustly elevated in cisplatin-treated mice (cp: 87.4 ± 3.3 versus CTR: 14.8 ± 0.4 mg/dL, *p* < 0.01). However, GYY4137 administration resulted in a greater elevation of BUN (104.6 ± 6.6 mg/dL, *p* < 0.01, versus cp group) (Figure 1(c)). Next, we studied the tubular injury via a PAS staining. Microscopically, the mice treated with cisplatin displayed severe pathological changes, characterized by the distortion of the overall renal morphology, dilation of renal tubules, and appearance of protein casts. Remarkably, these histological changes were more severe in GYY4137-pretreated animals (Figures 1(a) and 1(b)). These data suggested that this H_2_S donor played a detrimental role in cisplatin-induced nephrotoxicity.

### 3.2. GYY4137 Promoted Cisplatin-Induced Inflammation

**Figure 2:**
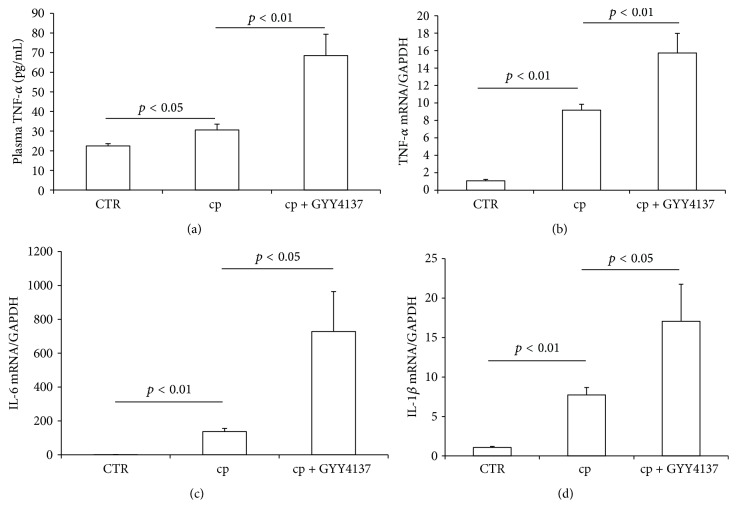
Effects of GYY4137 on cisplatin-induced inflammatory response. (a) Enzyme-linked immunosorbent assay analysis of circulating TNF-*α*. (b) qRT-PCR analysis of renal TNF-*α* mRNA expression. (c) qRT-PCR analysis of renal IL-6 mRNA expression. (d) qRT-PCR analysis of renal IL-1*β* mRNA expression. CTR: control, *n* = 5; cp: cisplatin, *n* = 10; and cp + GYY4137: cisplatin + GYY4137, *n* = 10. Data are means ± SE.

It is well known that the activation of inflammation was involved in the pathogenesis of cisplatin nephrotoxicity. Particularly, tumor necrosis factor-*α* (TNF-*α*) has shown a central role in mediating cisplatin-induced inflammation [17]. Therefore we measured the expression of renal TNF-*α* mRNA and circulating TNF-*α* level. As shown by the data, renal TNF-*α* mRNA was increased by 9.2-fold in cisplatin group as compared to the control mice, while GYY4137 pretreatment resulted in a much higher renal TNF-*α* mRNA expression than cisplatin alone group (Figure 2(b)). Consistent with the regulation of TNF-*α* in kidney, GYY4137 also promoted circulating TNF-*α* level after cisplatin treatment (Figure 2(a)). Meanwhile, the expressions of renal interleukin-6 (IL-6) and IL-1*β* mRNA showed similar patterns as renal TNF-*α* regulation (Figures 2(c) and 2(d)).

### 3.3. GYY4137 Aggravated Cisplatin-Induced Oxidative Stress

**Figure 3:**
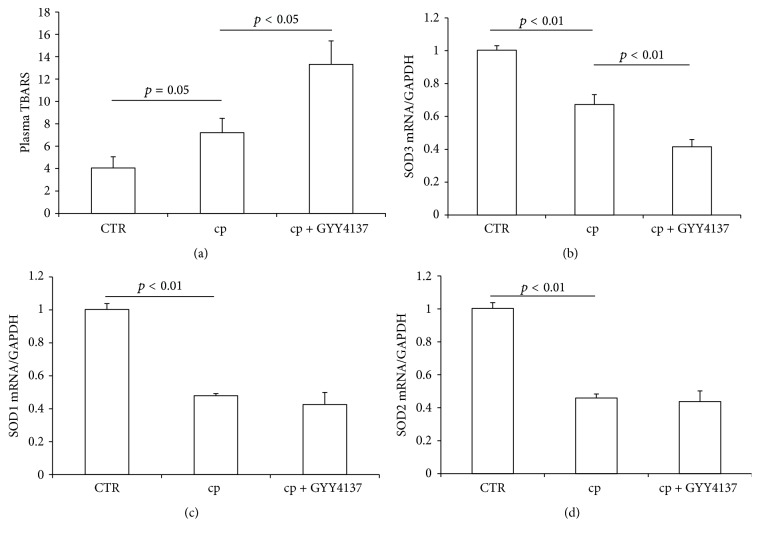
Effects of GYY4137 on cisplatin-induced oxidative stress. (a) Measurement of circulating thiobarbituric acid-reactive substances (TBARS) levels. (b) qRT-PCR analysis of renal SOD3 mRNA expression. (c) qRT-PCR analysis of renal SOD1 mRNA expression. (d) qRT-PCR analysis of renal SOD2 mRNA expression. CTR: control, *n* = 5; cp: cisplatin, *n* = 10; and cp + GYY4137: cisplatin + GYY4137, *n* = 10. Data are means ± SE.

Oxidative stress is another important factor responsible for cisplatin nephrotoxicity besides inflammation [18, 19]. Therefore we assessed the effect of GYY4137 pretreatment on cisplatin-induced oxidative stress. The level of plasma TBARS, a marker of oxidative stress, was increased from 4.1 ± 1.0 (control group) to 7.2 ± 1.3 (cisplatin alone group), while the TBARS level was further increased to 13.3 ± 2.1 in GYY4137 plus cisplatin group (Figure 3(a)). Moreover, we found that the cisplatin-induced oxidative stress was associated with the downregulation of antioxidative enzymes superoxide dismutase 1 (SOD1), SOD2, and SOD3, among which SOD3 was further decreased by GYY4137. These findings indicated that GYY4137 pretreatment aggravated cisplatin-induced oxidative stress possibly via a suppression of SOD3 following a challenge of cisplatin.

### 3.4. GYY4137 Aggravated Cisplatin-Induced Apoptotic Response in the Kidney

**Figure 4:**
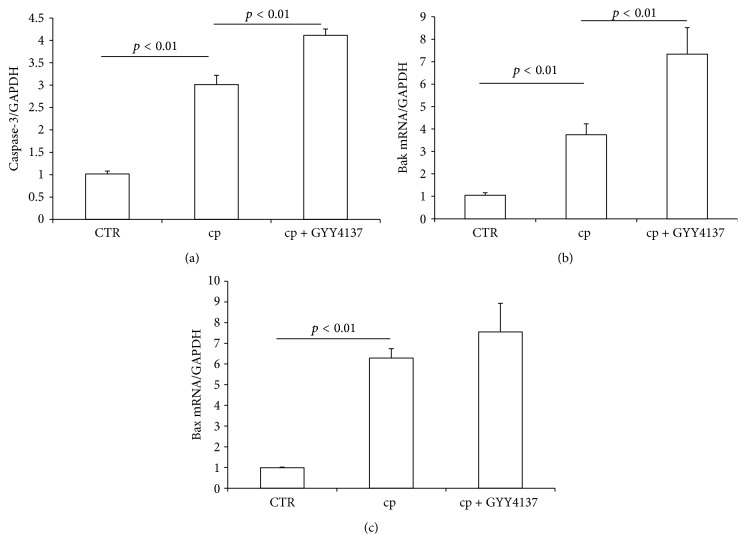
Effects of GYY4137 on cisplatin-induced apoptotic response. (a) qRT-PCR analysis of caspase-3 mRNA expression in kidney. (b) qRT-PCR analysis of Bak mRNA expression in kidney. (c) qRT-PCR analysis of Bax mRNA expression in kidney. CTR: control, *n* = 5; cp: cisplatin, *n* = 10; and cp + GYY4137: cisplatin + GYY4137, *n* = 10. Data are means ± SE.

Apoptotic pathway is also reported to be a molecular mechanism of cisplatin-induced nephrotoxicity, and the Bak and caspase activation served as potential key elements [20, 21]. Here we measured renal mRNA expression of caspase-3, Bak, and Bax in mice from different groups. We found the enhanced mRNA expressions of caspase-3 and Bak after cisplatin treatment were further increased in GYY4137-pretreated animals (Figures 4(a) and 4(b)). In contrast, the induction of Bax showed no difference between cisplatin alone group and GYY4137 plus cisplatin group (Figure 4(c)). Overall, these data suggested that GYY4137 could aggravate apoptotic response in cisplatin nephrotoxicity.

### 3.5. GYY4137 Treatment Had No Effect on the Downregulation of Renal CBS and CSE after Cisplatin Administration

**Figure 5:**
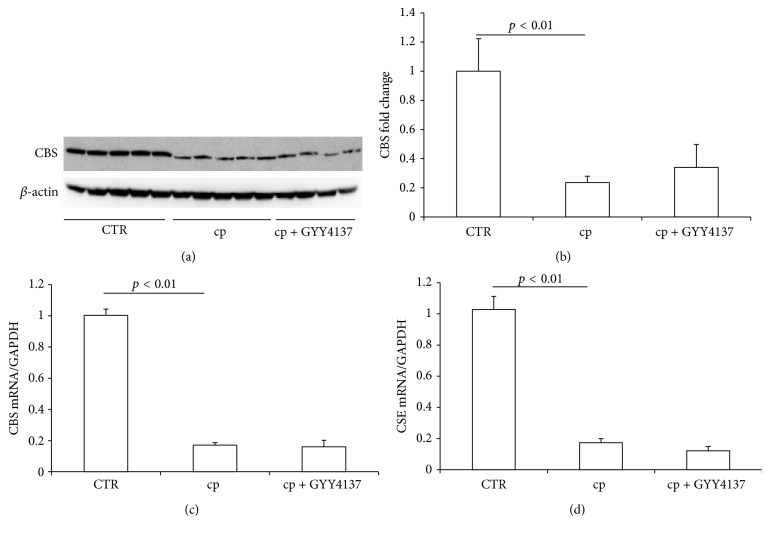
Effects of GYY4137 on CBS and CSE expression after cisplatin treatment. (a) Western blot analyses of CBS and *β*-actin in kidney. (b) Densitometry of CBS. A densitometric ratio between the densitometry of CBS and *β*-actin was calculated, and data are expressed in comparison with the controls. (c) qRT-PCR analysis of CBS. (d) qRT-PCR analysis of CSE. CTR: control, *n* = 5; cp: cisplatin, *n* = 10; and cp + GYY4137: cisplatin + GYY4137, *n* = 10. Data are means ± SE.

To demonstrate whether GYY4137 affected the expression of endogenous H_2_S-producing enzymes, we detected the mRNA and/or protein levels of CBS and CSE in the kidney. Both CBS (Figures 5(a)–5(c)) and CSE (Figure 5(d)) were significantly decreased after cisplatin treatment, which was unaffected by GYY4137. Thus the reduction of CBS and CSE might act as a protective mechanism against cisplatin-induced renal injury.

### 3.6. GYY4137 Alone Did Not Result in Renal Injury

**Figure 6:**
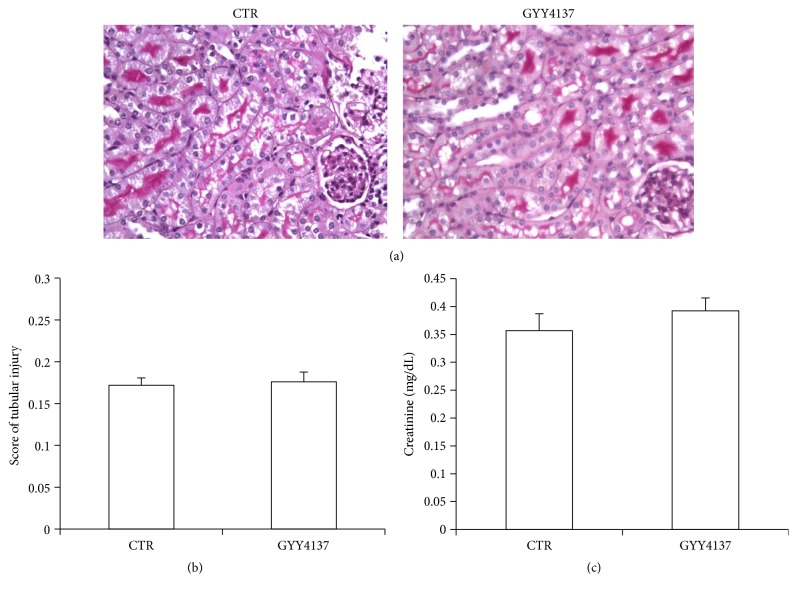
Effects of GYY4137 alone on renal morphology and function. (a) Representative images of periodic acid-Schiff staining (×400) of kidneys. (b) Tubular injury score. (c) Plasma creatinine levels. *n* = 5 in each group. Data are means ± SE.

To exclude the effect of the drug alone on renal structure and function, we set up the GYY4137 alone group and measured plasma creatinine and observed the morphological changes. The plasma creatinine level in GYY4137 group was not significantly different from that in CTR group (GYY4137: 0.392 ± 0.023 versus CTR: 0.357 ± 0.030 mg/dL, *p* > 0.05). Next, we studied the tubular injury via a PAS staining. Microscopically, the mice treated with GYY4137 did not display any pathological changes (Figures 6(a) and 6(b)). These data suggested that GYY4137 alone did not result in obvious nephrotoxicity.

## 4. Discussion

Hydrogen sulfide (H_2_S) has been known as a toxic gas with “rotten egg” smell for a long period of time. It shows its toxicity possibly via inhibiting mitochondrial cytochrome c oxidase (CcO) and oxidative phosphorylation and thus decreasing the production of adenosine triphosphate (ATP). However, growing evidence has demonstrated the biological and physiological importance of H_2_S. Its physiological relevance was firstly reported by Abe and Kimura where endogenous H_2_S serves as a neuromodulator in the brain and facilitates the hippocampal long-term potentiation by enhancing NMDA receptor-mediated responses [22]. In addition, H_2_S also displays important functions in cardiovascular system, kidney, liver, gastrointestinal, and endocrine systems [23–27]. In this regard, H_2_S has been widely acknowledged as the third gasotransmitter besides NO and CO. In kidney, H_2_S promotes urinary sodium excretion via both tubular and vascular mechanisms [25]. H_2_S can also inhibit renin-angiotensin system (RAS) by decreasing reactive oxygen species (ROS) generation [28] or cyclic adenosine monophosphate (cAMP) generation [29]. Meanwhile, H_2_S modulates renal oxidative stress response through upregulating antioxidant haem oxygenase-1 (HO-1) in human mesangial cells [30] and acts as an oxygen sensor in renal medulla [31]. Overall, accumulating evidence clearly demonstrated important roles of H_2_S in the kidney. Therefore, aberrant regulation of renal H_2_S might contribute to the pathogenesis of kidney diseases and thus modulation of endogenous or exogenous H_2_S could be potentially effective for treating renal diseases.

CBS and CSE are two major enzymes responsible for H_2_S production. Watanabe et al. reported that homozygous CBS mutants have about 40 times plasma homocysteine levels as normal and a majority of them died within 5 weeks after birth [32]. Mutant mice lacking CSE displayed pronounced hypertension in line with diminished endothelium-dependent vasorelaxation [33]. Decreased endogenous H_2_S production was reported to be associated with various diseases. The aortic H_2_S production was decreased by half in spontaneously hypertensive rats (SHRs) compared to normotensive rats and exogenous NaHS supplement attenuated hypertensive vascular collagen remodeling [34]. H_2_S deficiency also contributed to the progression of renal fibrosis. Endogenous H_2_S production was decreased during obstructive nephropathy and NaHS treatment attenuated unilateral ureteral obstruction- (UUO-) induced renal fibrosis [9, 35]. Moreover, recent report demonstrated that suppressed CSE/H_2_S pathway contributed to the pathogenesis of streptozotocin- (STZ-) induced DN [36]. Consistently, Zhou et al.'s results proved that H_2_S alleviated STZ-induced DN via attenuating oxidative stress and inflammation and inhibiting renin-angiotensin system activity [37].

Considering the anti-inflammatory and antioxidant effects of H_2_S, we tend to expect protective effects of H_2_S in kidney diseases. However, the facts are not that simple as expected. Inhibition of endogenous H_2_S formation by PAG (an irreversible inhibitor of CSE) could attenuate both cisplatin- and gentamicin-induced nephrotoxicities in rat models [13, 38–40], which suggested that endogenous H_2_S may aggravate kidney injury. In contrast, exogenous H_2_S donors, including NaHS and sodium thiosulfate (STS), were reported to protect against both cisplatin- and gentamicin-induced nephrotoxicities [10, 14, 41]. In consideration of these conflicting results, the role of H_2_S in kidney injury is hard to conclude and thus deserves further evaluation. In the present study, we employed a novel H_2_S donor, GYY4137, to study its role in cisplatin-induced nephrotoxicity. Our results showed that GYY4137 further exacerbated cisplatin-induced renal injury by aggravating inflammation, oxidative stress, and apoptosis in the kidney.

It is well accepted that inflammation is involved in the pathogenesis of cisplatin nephrotoxicity. Renal, circulatory, and urinary tumor necrosis factor-*α* (TNF-*α*) and other proinflammatory cytokines including interleukin 1*β* (IL-1*β*) were known to be upregulated by cisplatin injection [17, 42, 43]. TNF-*α* seemed to play a central role in the activation of inflammatory cascade since that inhibition of TNF-*α* via genetic or pharmacological approach strikingly attenuated cisplatin nephrotoxicity [17]. Our results showed a significant increase of circulating TNF-*α* and renal TNF-*α* mRNA expression after cisplatin administration. Meanwhile, renal IL-1*β* and IL-6 mRNA expression were remarkably increased in cisplatin group. These increments of inflammatory cytokines were further increased in cisplatin plus GYY4137 group, which indicated that GYY4137, a H_2_S donor, exacerbated the inflammatory response induced by cisplatin.

Oxidative stress is another known contributor of cisplatin-induced renal injury. Antioxidants are shown to be protective against cisplatin treatment both in cultured renal tubular cells [19, 44] and in animal models [45, 46]. Our results also showed an elevation of plasma TBARS level accompanied by significantly decreased renal expressions of SOD1, SOD2, and SOD3. Similar to GYY4137 effects on inflammation, this H_2_S donor aggravated oxidative stress in cisplatin-treated animals in line with a further reduction of SOD3. In agreement with promoted inflammation and oxidative stress, GYY4137 further enhanced the mRNA expressions of caspase-3 and Bak, suggesting that apoptotic response was also deteriorated.

Considering the aggravated effect of this H_2_S donor on cisplatin-induced renal injury, we detected the expression of endogenous H_2_S-producing enzymes in the kidney. The mRNA expressions of CBS and CSE were significantly decreased after cisplatin treatment, which was not affected by GYY4137. The protein level of CBS further confirmed this result. The previous study demonstrated the consistent results that GYY4137 increased the levels of H_2_S but had little effect on H_2_S-synthesizing activity [47]. The apparent downregulation of CBS and CSE probably would result in reduced H_2_S production, which might be a protective mechanism against cisplatin injury. In agreement with our findings, previous study showed that inhibition of endogenous H_2_S formation ameliorated the injury after cisplatin administration [13].

GYY4137 is a novel slow-releasing H_2_S donor while the conventional donors such as NaHS release H_2_S instantaneously in aqueous solution. Ahangarpour et al. reported that NaHS ameliorated the kidney dysfunction and damage in cisplatin-induced nephrotoxicity in Sprague-Dawley rats [14], which disagreed with our results. We think the difference of the species (mouse and rat) and/or H_2_S donors might result in the discrepancies. Bolus intravenous or intraperitoneal administration of GYY4137 to anesthetized rats increased plasma H_2_S concentration at 30 minutes and remained elevated during the 180-minute time course, while NaHS delivery to rats did not elevate plasma H_2_S levels [15]. Thus it is reasonable to expect that plasma H_2_S was increased and remained at a steady level in the current experiment with the use of microosmotic pump for GYY4137 delivery.

In summary, this study firstly examined the role of a novel exogenous H_2_S donor GYY4137 in a mouse model of cisplatin nephrotoxicity. Following cisplatin administration, the mice showed severe renal injury accompanied with increased oxidative stress, inflammation, and apoptotic response, which was further aggravated by GYY4137, suggesting a detrimental role of exogenous H_2_S in cisplatin-induced kidney injury in mouse.

## Figures and Tables

**Table 1:** Sequences of qRT-PCR primers.

Gene	Primer sequence	Accession number
*GAPDH*	5′-GTCTTCACTACCATGGAGAAGG-3′	M32599
	5′-TCATGGATGACCTTGGCCAG-3′	

*TNF-α*	5′-TCCCCAAAGGGATGAGAAG-3′	NM_013693
	5′-CACTTGGTGGTTTGCTACGA-3′	

*IL-6*	5′-CTTCCCTACTTCACAAGTCCGG-3′	NM_031168
	5′-GCCACTCCTTCTGTGACTCCAG-3′	

*IL-1β*	5′-ACTGTGAAATGCCACCTTTTG-3′	NM_008361
	5′-TGTTGATGTGCTGCTGTGAG-3′	

*SOD1*	5′-AAGGCCGTGTGCGTGCTGAA-3′	NM_921076
	5′-CAGGTCTCCAACATGCCTCT-3′	

*SOD2*	5′-CGGCCTACGTGAACAATCTC-3′	NM_013671
	5′-GATAGCCTCCAGCAACTCTCC-3′	

*SOD3*	5′-TTCTTGTTCTACGGCTTGCTAC-3′	NM_011435
	5′-CTCCATCCAGATCTCCAGCACT-3′	

*bak*	5′-CGCTACGACACAGAGTTCCA-3′	NM_007523
	5′-TCCATCTGGCGATGTAATGA-3′	

*bax*	5′-TGCAGAGGATGATTGCTGAC-3′	NM_007527
	5′-GATCAGCTCGGGCACTTTAG-3′	

*Caspase-3*	5′-ACAGCACCTGGTTACTATTC-3′	NM_009810
	5′-CAGTTCTTTCGTGAGCAT-3′	

*CBS*	5′-GGATTCCCCACATTACCACA-3′	NM_012522
	5′-CTGATGCGGTCCTTCACAC-3′	

*CSE*	5′-ACCTTTGGCTCTGGGTGCT-3′	NM_017074
	5′-TCCTGAAGTGTTTCTCCATCC-3′	
